# Genetic interaction implicates iRhom2 in the regulation of EGF receptor signalling in mice

**DOI:** 10.1242/bio.201410116

**Published:** 2014-11-13

**Authors:** Owen M. Siggs, Adam Grieve, Hongmei Xu, Paul Bambrough, Yonka Christova, Matthew Freeman

**Affiliations:** 1 Wellcome Trust Sanger Institute, Hinxton, Cambridge CB10 1SA, UK; 2 Dunn School of Pathology, South Parks Road, Oxford OX1 3RE, UK

**Keywords:** Rhomboid, iRhom, Rhbdf2, Amphiregulin, Mouse, TACE, ADAM17

## Abstract

iRhoms are closely related to rhomboid intramembrane proteases but lack catalytic activity. In mammals iRhoms are known to regulate the trafficking of TACE, the protease that cleaves the membrane bound inflammatory cytokine TNF. We have mapped a spontaneously occurring mouse mutation with a loss of hair phenotype, *curly bare* (*cub*), to the *Rhbdf2* locus, which encodes the iRhom2 protein. The *cub* deletion removes the first 268 amino acids of the iRhom2 protein but is not a loss of function. We have also identified a previously reported suppressor of *cub*, called *Mcub* (*modifier of curly bare*), and find it to be a loss of function allele of the amphiregulin gene (*Areg*). Amphiregulin is an activating ligand of the epidermal growth factor receptor (EGFR) that, like TNF, is released by TACE. Our results therefore imply a regulatory link between iRhoms and EGFR signalling in mammals. We have tested the model that the *cub* mutation leads to iRhom2 hyperactivity and consequently excess TACE processing of amphiregulin and elevated EGFR signalling. Our results do not support this hypothesis: we find that, compared to wild-type cells, *cub* mutant embryonic fibroblasts release less amphiregulin, and that the *cub* mutant form of iRhom2 is less able than wild type to bind to TACE and promote its maturation.

## INTRODUCTION

The earliest members of the rhomboid-like superfamily to be discovered were the rhomboid intramembrane serine proteases (Urban et al., 2001) which, by cleaving transmembrane helices and releasing soluble domains, have multiple functions in controlling signalling and other cellular functions (Urban and Dickey, 2011). More recently, the extent of the rhomboid-like superfamily has become clear, and this has highlighted that many members of this clan are predicted not to be active proteases (Freeman, 2014). The most studied non-protease rhomboid-like proteins are the iRhoms, which regulate trafficking and degradation of client proteins (Adrain et al., 2012; Maretzky et al., 2013; McIlwain et al., 2012; Siggs et al., 2012; Zettl et al., 2011). Despite intense recent scrutiny, there are still many aspects of the physiological role of iRhoms that are poorly understood.

Both in *Drosophila* and mammals, a genetic approach has underpinned our understanding of iRhom function: loss of function mutations in flies and mice have revealed the role of iRhoms in both ER associated degradation, and the control of trafficking of the metalloprotease TACE, the enzyme that releases active TNF and ligands of the EGF family (Adrain et al., 2012; McIlwain et al., 2012; Zettl et al., 2011). Beyond these simple loss of function alleles, disease mutations in humans have also been isolated. Several groups have shown that a rare familial hyperkeratosis and oesophageal cancer syndrome, tylosis, is caused by missense mutations in specific locations within the cytoplasmic N-terminus of iRhom2 (Blaydon et al., 2012; Saarinen et al., 2012); these have also been associated with ovarian cancer (Wojnarowicz et al., 2012). The molecular effect of these mutations is not yet clear, but they identify an important functional site in the iRhom2 protein, and there is recent evidence that they lead to increased release of EGF family ligands in keratinocytes (Brooke et al., 2014).

Johnson et al. reported a recessive mouse mutation on chromosome 11 with a hair-loss phenotype that they named *curly bare* (*cub*) (Johnson et al., 2003). The *cub* mutation mapped to an interval that included *Rhbdf2*, the gene encoding iRhom2. In the same work, it was shown that a second site dominant suppressor of the *cub* phenotype existed on chromosome 5; this was called *Mcub* for modifier of *cub*. One copy of the *Mcub* allele was sufficient to rescue the hair loss of *cub*/*cub*, although the coat of these rescued mice was wavy. Significantly, wavy coat phenotypes are characteristic of mutations in the EGFR pathway (Schneider et al., 2008).

Here we report that the *cub* mutation is a deletion of the N-terminal cytoplasmic domain of the *Rhbdf2* gene, which encodes iRhom2. Mice with complete loss of iRhom2 have normal coats (Adrain et al., 2012; McIlwain et al., 2012), implying that the *cub* mutation is not a simple loss of function. We have also used whole genome sequencing to identify the modifier mutation on chromosome 5 as being a loss of function variant of the amphiregulin gene, *Areg*. Amphiregulin is a ligand for the EGFR, strengthening the case for functional links between iRhom2 and EGFR signalling. These results are consistent with two very recent reports, one of which also identifies the *cub* and *Mcub* mutations (Hosur et al., 2014), and the other which reports that a distinct but overlapping deletion in the cytoplasmic domain of iRhom2 leads to another mouse hair-loss phenotype (Liu et al., 2014). We have further explored the mechanistic implications of the links between iRhom2 and amphiregulin and we report here that the simple interpretation that the *cub* mutation is a gain of iRhom2 function, causing excess amphiregulin release, is not fully supported. The actual relationship between iRhom2, amphiregulin and EGFR signalling is more complex.

## MATERIALS AND METHODS

### Genome sequencing

Tail DNA from the *cub;Mcub* strain (003628 B6.Cg-*Mcub cub*/J) was obtained from the Jackson Laboratory, and subjected to whole-genome sequencing as described (Bull et al., 2013). Briefly, 100 bp paired-end libraries were prepared and sequenced on a single lane of an Illumina HiSeq 2000. Reads were aligned to the mouse reference genome (mm10) using Stampy (Lunter and Goodson, 2011) with BWA settings, and duplicate reads were identified and discarded using the MarkDuplicates tool (http://broadinstitute.github.io/picard). 94.6% of the reference genome was covered at least three times (as determined by BEDtools). Variants were called by Platypus version 0.1.9 (Rimmer et al., 2014), and annotated using ANNOVAR (Wang et al., 2010) with RefSeq and dbSNP 138 annotations.

All animal procedures in this manuscript were performed in compliance with the institutional animal care committee guidelines of the University of Oxford and according to protocols approved by the UK Home Office.

### Antibodies

The following antibodies were used: Goat anti-mouse amphiregulin (R&D Systems, catalogue number AF989; 1:1000), Mouse anti-nonphospho-Tyr1173-EGFR (Millipore, catalogue number 05-484; 1:1000), mouse anti-beta-actin (Santa Cruz, catalogue number sc-47778; 1:2000), rabbit anti-TACE/ADAM17 (Abcam, catalogue number ab39161; 1:2000), rabbit anti-HA (Santa-Cruz, catalogue number sc-805; 1:2000) and mouse anti-transferrin receptor (Invitrogen, catalogue number 13-6800; 1:1000). Corresponding species-specific HRP-coupled secondary antibodies were used from Santa Cruz and Cell Signaling.

### Molecular biology

pM6P.blast-GFP was a kind gift from Felix Randow (LMB, Cambridge). For pM6P.blast iRhom2-3xHA and pM6P.blast *cub*-3xHA, corresponding coding sequences were PCR amplified, the plasmid backbone was digested with restriction enzyme sites NcoI/XhoI and NcoI/NotI, respectively and PCR products were inserted using the InFusion cloning system (Clontech). All plasmids were verified by DNA sequencing.

### Cell culture

Mouse embryonic fibroblasts (MEFs) were isolated from C57BL/6J *cub/cub* E13.5 embryos and their wild-type (WT) littermates, and immortalised by lentiviral transduction with SV40 large T antigen. All cells used were maintained in regular high-glucose DMEM, supplemented with 10% FCS, 100 µg/ml penicillin, and 100 µg/ml streptomycin.

### TCA precipitation

For analysis of amphiregulin secretion, MEFs were plated in 35 mm dishes and grown to 100% confluency, then incubated in 1.5 ml serum-free medium for 24 hrs. After medium was removed, clarified by sedimentation at 800 × g, and the resulting protein in the supernatant was precipitated by incubation with trichloroacetic acid (TCA) as previously described (Adrain et al., 2011).

### AP-shedding assay

To test stimulated amphiregulin (Areg) or EGF (used as a control) shedding, MEFs were plated at a density of 1×10^5^ per well of a 24 well plate followed by transfection 24 hours later with alkaline phosphatase (AP) conjugated Areg or EGF (kind gifts of Carl Blobel). For transfection, 200 ng DNA and 0.9 µl of Fugene-6 (Promega) were used, following standard protocols. One day later a stimulation assay was performed as described previously (Christova et al., 2013). In short, cells were washed twice in PBS and incubated in 200 µl Opti-MEM (Invitrogen) for 1 hour. Cells were then stimulated by the addition of supra-maximal concentrations of PMA (100 nM to 1 µM) for 1 hour. AP activity was detected in stimulated and un-stimulated supernatant or in cell lysates (using Triton X-100 buffer) by adding equal volumes of PNPP buffer (Pierce) and incubating at 37°C followed by measurement of absorbance at 405 nm. The percentage of the total material shed from each well (i.e. signal from supernatant divided by total signal from lysate and supernatant) was then used to calculate the percentage-stimulated induction.

### Retroviral transduction of iRhom1;iRhom2 double knock-out (DKO) MEFs

To generate retrovirus, a pCL-based retrovirus packaging system was used. HEK293 cells grown to 80% confluence were transfected with Lipofectamine in 35 mm plates with 1 µg of the expression plasmid (termed pM6P.blast-GFP, pM6P.blast iRhom2-3xHA and pM6P.blast *cub*-3xHA) and 1 µg of the packaging plasmid pCL-Eco. The following day, medium was changed and transfected cells were allowed to secrete virus for 20 hours in 2 ml of medium. Culture supernatants were then filtered centrifuged at 20,000 × g, to clear.

For infection of target iRhom1;iRhom2 double knock-out (DKO) cells, viral supernatants were diluted 2-fold in fresh medium for transduction. Transduction was carried out in the presence of 50 µg/ml polybrene and a medium change was made 24 hours later. For selection of pM6P plasmids, cells were treated with 5 µg/ml blasticidin 48 hours later.

### Lentiviral transduction of HEK293 cells

Using the pLex-based system (Thermo Scientific) for lentiviral production, HEK293 cells were transduced in the following way. Cells were plated on 6 cm plates one day before transfection, then transfected with 1 µg LTR plasmid containing iRhom2-3xHA or *cub*-3xHA, 0.7 µg pCMV delta8.91 (packaging plasmid) and 0.3 µg VSVG (envelope) in serum-free medium and PEI. After 24 hours, medium was removed and replaced with high-glucose DMEM, supplemented with 10% FCS, 100 µg/ml penicillin, and 100 µg/ml streptomycin. After 72 hours, medium was collected and centrifuged at 1,000 × g. Viral preparations were combined with 50 µg/ml polybrene and added to fresh HEK293 cells. After 24 hours, pLex-positive cells were washed and selected with 6 µg/ml puromycin for 7 days before assaying.

### Immunoprecipitation and ConA enrichment

For ConA enrichment experiments, cells grown in 10 cm dishes were grown to ∼80% confluence were washed twice in ice-cold PBS and then lysed for 10 minutes in TX-100 lysis buffer (1% Triton X-100, 150 mM NaCl, 50 mM Tris-HCl, pH 7.4, supplemented with 1 mM EDTA, 1 mM CaCl^2+^ and 1 mM MnCl^2+^) containing complete protease inhibitor cocktail (Roche) and 10 mM 1,10-phenanthroline, to prevent autocatalysis of TACE. Cells/lysates were then scraped and centrifuged at 20,000 × g. Protein concentration was then measured using BCA kit.

Lysates were then mixed with washed 50 µl ConA beads (washed in lysis buffer) and incubated for 2–3 hours at 4°C on a rotor. Beads were washed 4–5 times in lysis buffer and glycoproteins were eluted with 1× sample buffer supplemented with 15% sucrose, by incubation at 65°C for 15 mins in a thermomixer. 20% of the ConA preparations and 1% of lysates were resolved on SDS-PAGE gels for subsequent western blotting.

For immunoprecipitations, HEK293 cells stably transduced with iRhom2-HA or *cub*-HA were seeded at 8×10^6^ cells per 10 cm plate and cultured overnight. After stimulation with or without 200 nM PMA for 15 mins, the cells were washed with ice-cold PBS and then lysed in 1 ml TX-100 lysis buffer (1% Triton X-100, 150 mM NaCl, 50 mM Tris-HCl, pH 7.4) supplemented with protease inhibitor cocktail (Roche) and 10 mM 1,10-phenanthroline. Cell lysates were cleared by centrifugation at 20,000 × g for 20 mins at 4°C. Protein concentrations were measured by a BCA assay kit (Pierce). The lysates were then immunoprecipitated for 2–3 hours with 15 µl pre-washed HA antibody-coupled beads at 4°C on a rotor. After 4–5 washes with lysis buffer, the immunocomplexes were incubated at 65°C for 15 mins in 1× sample buffer. 20% of the immunoprecipitates and 1% of lysates were resolved on SDS-PAGE gels for subsequent western blotting.

### SDS-PAGE and western blotting

Samples were typically electrophoresed at 200 V on 4–12% Bis-Tris gels (Invitrogen) until the dye front had migrated off the gel (approx. 10–15 kDa). Gels were transferred onto PVDF membranes and blocked in PBS or TBS containing Tween 20 (0.05%) and 5% milk, before detection with the indicated primary antibodies and species-specific HRP-coupled secondary antibodies. Band visualisation was achieved with Enhanced Chemiluminescence (Amersham Biosciences) using X-ray film.

## RESULTS AND DISCUSSION

### Identification of the genes mutated in *cub* and *Mcub* mice

**Fig. 1.**
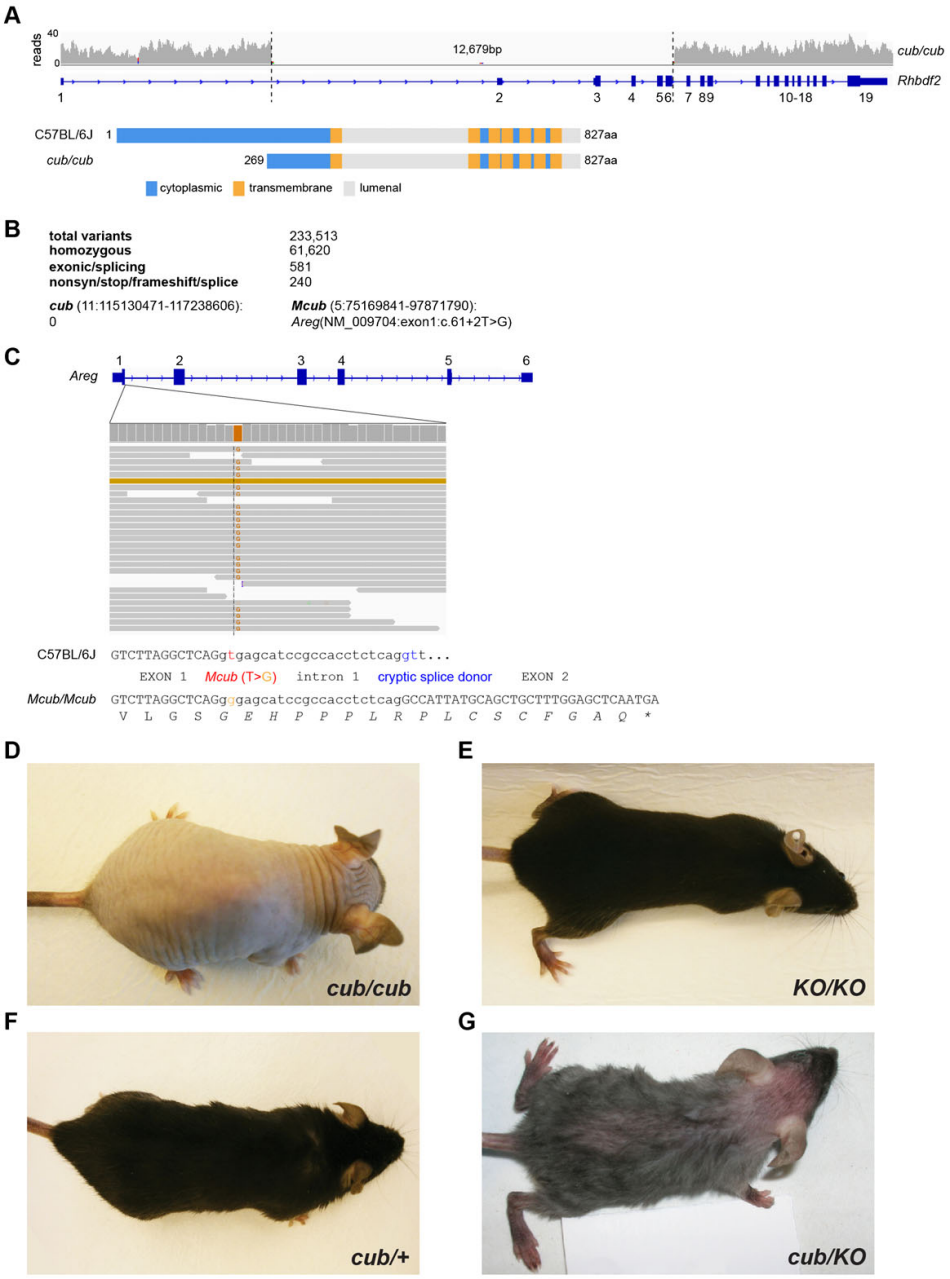
*cub* and *Mcub* are mutations at the *Rhbdf2* and *Areg* loci, respectively. (A) Coverage depth across the *Rhbdf2* locus in *cub/cub* mice, and predicted translational consequences of the *cub* deletion on iRhom2 protein. (B) Filtering pipeline for variants detected after whole-genome sequencing of *cub/cub;Mcub/Mcub* DNA. (C) Identification and predicted consequences of the *Areg^Mcub^* variant. (D) The bald phenotype of an *Rhbdf2^cub^/Rhbdf2^cub^* mouse (Johnson et al., 2003). (E) *Rhbdf2^KO^/Rhbdf2^KO^* mouse has a WT coat (Adrain et al., 2012; McIlwain et al., 2012). (F) *Rhbdf2^cub^* is recessive: *Rhbdf2^cub^*/+ mouse has a WT coat (Johnson et al., 2003). (G) *Rhbdf2^cub^/Rhbdf2^KO^* mouse has sparse hair (Hosur et al., 2014). Mice in panels D–G are on a C57BL/6J background, except for *Rhbdf2^cub^/Rhbdf2^KO^*, which is on a mixed C57BL/6J/129 background.

To identify the nature of the *cub* and *Mcub* mutations (Johnson et al., 2003), we sequenced the genome of a *cub*/*cub;Mcub/Mcub* double homozygote. *cub* arose spontaneously within an inbred colony of urogenital syndrome (*us*) mice at the Jackson Laboratory (*a/a us/us*), and has previously been mapped to a 75-gene interval between D11Mit214 and D11Mit303 (chr11:115130471–117238606) (Johnson et al., 2003). Interestingly, a similar phenotype, *Uncovered*, has also been mapped between D11Mit338 and D11Mit337 (chr11:115461783–118997798) (Shi et al., 2003). Although we detected no single nucleotide variants or short indels in the *cub* interval, a large deletion was present at the *Rhbdf2* locus (Fig. 1A). The *Rhbdf2^cub^* deletion spanned 12,679 nucleotides (chr11: 116604896–116617574, inclusive), which included exons 2–6. This deletion is predicted to result in the splicing of exon 1 to exon 7, the initiation of translation from an in-frame methionine codon in exon 8, and therefore the loss of the first 268 N-terminal residues, which include almost the entire cytoplasmic domain of iRhom2 (Fig. 1A).

Continuing our analysis of the *cub/cub;Mcub*/*Mcub* double homozygote, we sought to identify the *Mcub* mutation. From a total of 233,513 variants called across the genome, 240 were both homozygous and predicted to alter protein coding sense (i.e. nonsynonymous, stop, frameshift or splice variants) (Fig. 1B). Only one of these variants lay within the previously defined *Mcub* interval (chr5:75169841–97871790). This variant was a predicted critical splice donor mutation in the *Areg* gene (NM_009704:exon1:c.61+2T>G), and confirmed to be absent from CAST/EiJ, C3H/HeJ, and A/J inbred strains as established previously (Johnson et al., 2003). Given that the variant occurs after the first (coding) exon of *Areg*, we predicted that *Areg^Mcub^* would lead to intronic read-through, introduction of a premature termination codon in exon 2, and ultimately to nonsense-mediated decay (Fig. 1C).

These data establish that the hair defects seen in *cub*/*cub* homozygotes (Fig. 1D) are caused by a loss of the N-terminal cytoplasmic domain of iRhom2; that this mutation is not equivalent to a loss of function of iRhom2, which has a normal coat (Fig. 1E); and that the phenotype is largely suppressed by loss of one or both copies of the amphiregulin (*Areg*) gene. Our data agree with the recent identification of the *cub* and *Mcub* alleles by Hosur et al. (Hosur et al., 2014). Both reports are also consistent with the recent discovery of another hairless allele of *Rhbdf2*, which is also caused by deletion of a significant part of the iRhom2 cytoplasmic domain (Liu et al., 2014).

### Investigating the relationship between iRhom2 and amphiregulin

**Fig. 2.**
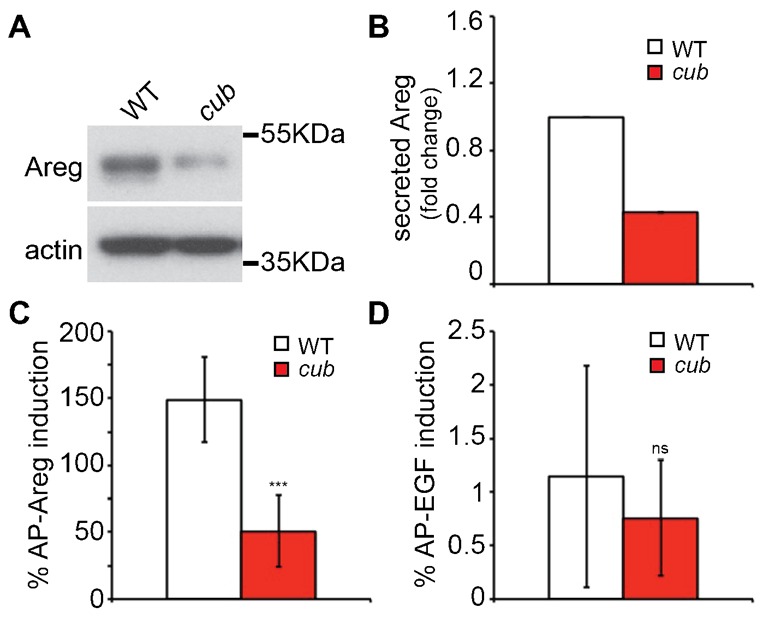
Constitutive and stimulus-induced amphiregulin release is reduced in *cub* MEFs. (A) Western blot analysis of soluble amphiregulin levels in medium precipitated from MEFs derived from *cub* mice or their WT littermates (C57BL/6J), grown in serum free conditions for 24 hours. Actin is shown as a control for loading. (B) Quantification of the reduction of amphiregulin secretion in *cub* MEFs, relative to WT MEFs. All data are normalised to actin levels. Data represent at least n = 3 individual experiments. (C) Shedding of alkaline phosphatase conjugated amphiregulin (AP-Areg) after stimulation with PMA for 1 hour. P values represent the result of a Student's t-test; AP-Areg WT versus *cub* comparison  =  2.53E−09. (D) Shedding of alkaline phosphatase conjugated epidermal growth factor (AP-EGF). P values represent the result of a Student's t-test; AP-EGF WT versus *cub* comparison: 0.48. All error bars represent standard deviation.

iRhoms play an essential role in the maturation of TACE (also named ADAM17), the enzyme responsible for the release of active amphiregulin (Gschwind et al., 2003; Hinkle et al., 2004). Given that the *cub* mutation is suppressed by a reduction of amphiregulin, we speculated that there would be excessive amphiregulin release from *cub* mutant cells. This would imply that *cub* represents a gain of function in iRhom2, which would be consistent with it having a hair-loss phenotype not seen in iRhom2 null mice. We therefore investigated the effect that the *cub* mutation has on iRhom2 function. First, we compared the ability of MEFs from *cub* and WT embryos to shed amphiregulin. To our surprise, instead of increased release, *cub* cells showed strongly reduced levels (approx. 60% compared to WT cells) of amphiregulin in the medium (Fig. 2A,B).

Since the ADAM family of proteases exhibit stimulus dependent as well as constitutive sheddase activity (Horiuchi et al., 2007), we separately examined the ability of *cub* mutant cells to promote the stimulated shedding of amphiregulin in a widely used assay (Zheng et al., 2002). MEFs were treated with PMA for one hour and the stimulated level of alkaline phosphatase (AP) tagged amphiregulin released into the medium was compared between WT and *cub* MEFs. The *cub* MEFs showed significantly reduced stimulated shedding of amphiregulin (Fig. 2C), demonstrating that both aspects of shedding activity (stimulated as well as constitutive) are diminished by the *cub* mutation. In comparison, there was no significant difference between WT and *cub* cells in their ability to shed AP-EGF, which does not depend on TACE for its release (Fig. 2D).

**Fig. 3.**
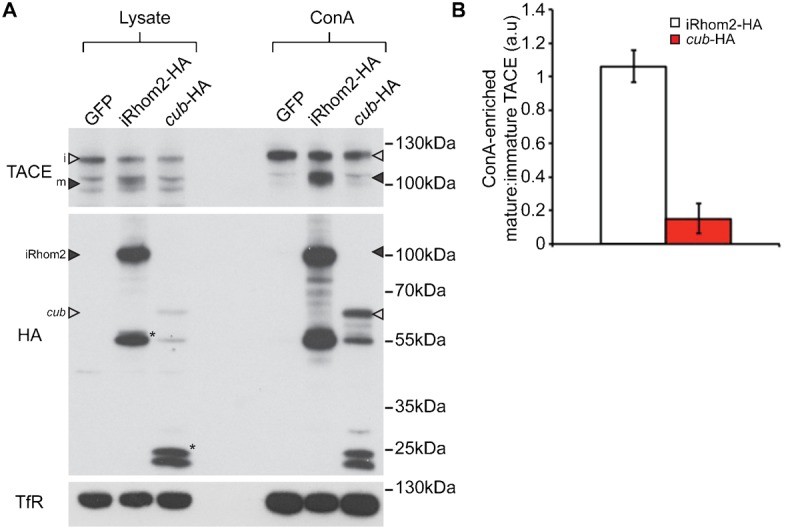
TACE maturation is not promoted by the *cub* mutant of iRhom2. (A) Western blot of TACE from lysates and ConA enrichment preparations from iRhom1/2 double knock-out MEFs transduced with GFP, iRhom2-HA or the *cub* mutant of iRhom2-HA. Levels of the transferrin receptor (TfR) were used as a loading control. Note that only iRhom2-HA promotes the maturation of TACE. In the blot for TACE, immature TACE is indicated with an empty arrowhead (i), mature TACE with a filled arrowhead (m). In the blot for HA, full-length iRhom2-HA is indicated with a filled arrowhead, and full-length *cub*-HA is indicated with an empty arrowhead. Asterisks (*) indicate sub-fragments of iRhom2-HA or *cub*-HA regularly observed on western blot. (B) Densitometric quantification of the ability of iRhom2-HA and *cub*-HA to promote the maturation of TACE in iRhom1/2 double knockout MEFs. Data represent the ratio of mature to immature TACE, relative to iRhom1/2 double knockout MEFs transduced with GFP (in which TACE maturation is blocked). n = 4 individual experiments. Error bars represent standard deviation.

iRhom2 is an essential factor in the trafficking and maturation of TACE, so we analysed the effect of the *cub* mutation on the ability of iRhom2 to promote TACE maturation (Fig. 3A). Double knockout MEFs, lacking both iRhom2 and the related iRhom1 were stably transduced with either WT or the *cub* mutant form of iRhom2-HA. Relative levels of endogenous immature and mature TACE were compared, before and after ConA enrichment (to concentrate glycoproteins from lysates). Consistent with the reduced release of amphiregulin, MEFs transduced with the *cub* mutant form of iRhom2 had substantially less mature TACE than WT counterparts (Fig. 3A,B).

**Fig. 4.**
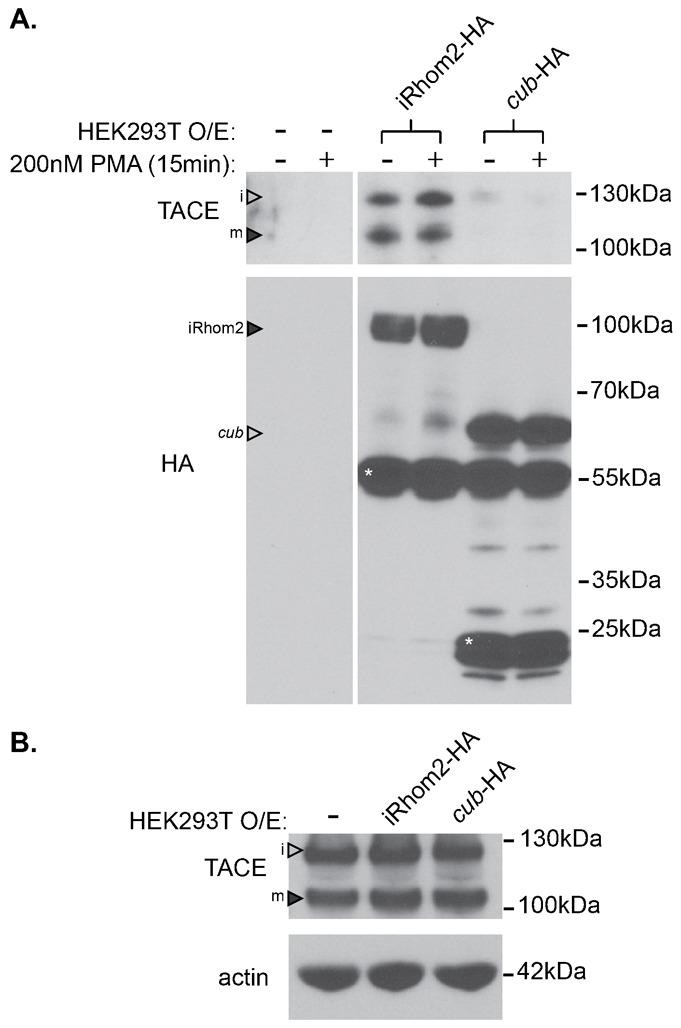
The *cub* mutation impairs iRhom2 binding to TACE. (A) Western blot analysis of HA-immunoprecipitates from HEK293 cells transduced with empty vector, iRhom2-HA or *cub*-HA and probed for binding to endogenous TACE. Cells were treated with or without 200 nM PMA, to induce shedding for 15 mins, but this had no effect on binding to TACE. (B) Western blot analysis of ConA concentrated samples from HEK293 cells transduced with empty vector, iRhom2-HA or *cub*-HA and probed for endogenous TACE and beta-actin. In the blots for TACE, immature TACE is indicated with an empty arrowhead (i), mature TACE with a filled arrowhead (m). In the blot for HA, full-length iRhom2-HA is indicated with a filled arrowhead, and full-length *cub*-HA is indicated with an empty arrowhead. Asterisks (*) indicate sub-fragments of iRhom2-HA or *cub*-HA regularly observed by western blot.

Finally, to test whether the reduced ability of the *cub* mutant form of iRhom2 to promote TACE maturation and amphiregulin shedding was caused by impaired binding between the *cub* mutant form of iRhom2 and its client, TACE, we stably expressed HA-tagged iRhom2 and the *cub* mutant form in HEK293 cells. Compared to WT iRhom2-HA, co-immunoprecipitation showed that the *cub* mutant form of iRhom2 showed greatly reduced binding to endogenous TACE (Fig. 4A). Analysis of levels of immature and mature TACE in these stable HEK293 cells revealed there is no major difference in TACE expression upon introduction of these constructs into the cell (Fig. 4B). These data demonstrate that the interaction between iRhom2 and TACE is strongly diminished by the *cub* mutation.

These results are consistent with each other but not with the model that the *cub* mutation causes a simple gain of function of iRhom2. In that case we would expect to see increased binding to and/or increased maturation of TACE, and elevated amphiregulin release. In each case our result is the opposite of that prediction. The genetics of *cub*, however, are complicated. First, we can rule out *cub* being a simple loss or reduction of iRhom2 function, since the complete loss of iRhom2 does not cause the coat defects seen in *cub* (compare Fig. 1D and Fig. 1E) (see also Adrain et al., 2012; McIlwain et al., 2012). Second, we can also rule out *cub* being a simple gain of function mutation because the hairless phenotype is fully rescued by a WT allele of iRhom2 but only somewhat rescued by a loss of function allele (compare Fig. 1F and Fig. 1G) (see also Hosur et al., 2014). In other words, addition of extra iRhom2 function (the WT allele in *cub*/+ compared to cub/iRhom2 KO) rescues the effect of the *cub* mutation – which is inconsistent with *cub* being a gain of function. Therefore *cub* appears to be a complex allele that combines gain and loss of function characteristics. It is also worth noting that most cells express iRhom1 as well as iRhom2 and their function appears at least largely interchangeable (Christova et al., 2013), so even in *cub* homozygous mice, there remains WT iRhom function.

We note that our failure to detect excess amphiregulin release induced by the *cub* mutant form of iRhom2 disagrees with the recent model proposed by Hosur et al., in which they show excess amphiregulin production in both *cub* serum and the supernatant of *cub* keratinocytes (Hosur et al., 2014). We now discuss some of the possible explanations for these significant discrepancies but concede that, while their model that *cub* represents a gain of function is not well supported by current data, we are not currently in a position to propose a well-supported alternative. One explanation to consider is that iRhom2 functions in a hair follicle-specific protein complex and that the *cub* mutation causes a tissue specific dominant negative effect. Although this would formally be consistent with all current data, there is no evidence for such a complex and the idea is purely speculative.

A striking result reported by Hosur et al. is that amphiregulin mRNA expression is elevated 4-fold in *cub* skin (Hosur et al., 2014). This result could provide an additional or alternative explanation for the increased amphiregulin production in *cub* mutant cells that they observe: instead of the *cub* mutant form of iRhom2 directly triggering amphiregulin release, the effect could be indirect, via a transcriptional response.

**Fig. 5.**
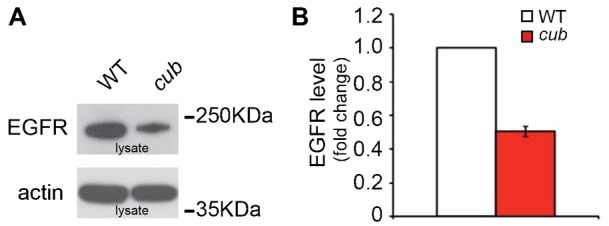
Levels of the EGFR are reduced in *cub* MEFs. (A) Western blot analysis of EGFR level in lysates from either WT or *cub* MEFs (C57BL/6J) in normal growth conditions. Actin is shown as a control for loading. (B) Quantification of the reduction in EGFR level in *cub* MEFs, relative to WT MEFs. All data are normalised to differences in actin levels. Data represent at least n = 3 individual experiments.

We noticed that in *cub* mutant MEFs there was a dramatic down-regulation of EGFR levels (Fig. 5A,B). This was also reported by Hosur et al. (Hosur et al., 2014). They interpreted this to be caused by increased endocytic degradation of cell surface receptor induced by the excess amphiregulin signalling. Indeed, EGFR levels are known to be tightly regulated by receptor activity (Wiley, 2003). However, this interpretation cannot easily be reconciled with our results since, in our case, the receptor downregulation occurs in cells with reduced, not increased, amphiregulin secretion.

Of relevance to this discussion, Brooke et al. have recently analysed disease mutations in the cytoplasmic domain of human iRhom2 (Brooke et al., 2014) and, reminiscent of the work of Hosur et al. (Hosur et al., 2014), they find that these mutations cause excess production of some EGF family ligands, including amphiregulin. But in contrast to Hosur et al., this was reported to be fully dependent on TACE activity. Hosur et al. are not explicit about which protease they believe to be responsible for elevated amphiregulin release in their experiments, but they find it to be insensitive to marimastat, an inhibitor of TACE and other ADAM proteases. They therefore conclude that the *cub* mutant form of iRhom2 promotes amphiregulin release by an ADAM protease-independent mechanism, a very different mechanistic conclusion to that of Brooke et al. (Brooke et al., 2014). In fact, Hosur et al. appear to favour the idea that there could be intrinsic proteolytic activity in the iRhom2 protein itself. This is a suggestion that is difficult to disprove formally but that would radically alter current models of iRhom function (Freeman, 2014): iRhom2 lacks all the necessary catalytic residues of an active rhomboid, indeed it has an activity-killing proline inserted at the site equivalent to a catalytic site in rhomboids. Moreover, it has never been found to cleave any potential substrate, and Hosur et al. do not support their suggestion with direct evidence to counter these earlier studies. On the current balance of evidence, therefore, we do not think that the model proposed by Hosur et al. is likely to be correct.

Having tried to be explicit about where our work differs from that of Hosur et al. (Hosur et al., 2014) (or where we believe their interpretations are inconsistent with other existing data), we wish to point out that the experimental differences, primarily whether amphiregulin release is decreased or increased, may be due to differences in how the experiments were performed. For example, in our work, we have used embryonic fibroblasts derived from *cub* embryos; Hosur et al. focused mainly on keratinocytes. Since the *cub* phenotype affects specifically hair follicle cells, neither MEFs nor keratinocytes are fully appropriate model cells, although it is surprising that they appear to give such opposing results. It is worth noting that the Kelsell lab, when studying the tylosis mutations in human cells (Brooke et al., 2014), also use keratinocytes but report results that, as described above, are not readily reconcilable with Hosur et al. (Hosur et al., 2014).

### Concluding remarks

Since our genetic identification of *cub* and *Mcub* exactly agrees with the recent report by Hosur et al. (Hosur et al., 2014), it is very surprising that our attempts to provide a mechanistic explanation differ so profoundly. While we disagree with many of their conclusions, we readily concede that we do not have a clear alternative picture. Further work will be needed to address these questions fully. We also want to emphasise that we do not rule out the possibility that amphiregulin overproduction is relevant to the *cub* phenotype. Indeed, the basic genetics supports this view, and other evidence, such as the ability of *cub* to enhance tumour growth in the *Apc^Min^* mouse model (Hosur et al., 2014), is consistent with the idea. However, the mechanistic explanation proposed by Hosur et al. is not easy to reconcile with current data. It is relevant to emphasise that we understand little about the cellular mechanisms by which iRhoms act. For example, there is no solid evidence for their physiological role in regulating ERAD in mammalian cells, despite it being an important function in *Drosophila* (Zettl et al., 2011). And if, in fact, iRhoms do have a role in mammalian ERAD, there is no understanding about how this relates to their function in forward trafficking of TACE.

We have shown that the *cub* mutation is a deletion predicted to cause the loss of 268 N-terminal amino acids of mouse iRhom2, leading to the production of a protein with most of its cytoplasmic domain deleted. We have also demonstrated that *Mcub*, the dominant suppressor of *cub*, is a loss of function mutation in the amphiregulin gene. These genetic data make a clear case for a functional relationship between iRhom2 function and EGFR signalling. This is certainly the case in *Drosophila*, where iRhom regulates EGFR signalling (Zettl et al., 2011). Importantly, though, in that case iRhom does not, as in mice, promote shedding but instead inhibits ligand production by degrading EGFR ligands as they pass through the secretory pathway. As noted above, we do not currently know the significance of these apparently profound differences between *Drosophila* and mammalian iRhom function. Our attempts to interpret mechanistically our current data to understand more about the role of iRhom2 in EGFR signalling in mice have failed to lead to a clear model. Contrary to the superficially attractive idea that *cub* is a gain of function allele of iRhom2, we find amphiregulin release to be reduced in *cub* MEFs, TACE maturation to be impaired, and the binding of the *cub* mutant form of iRhom2 to its client TACE also to be reduced.
